# Designing an optimized diagnostic network to improve access to TB diagnosis and treatment in Lesotho

**DOI:** 10.1371/journal.pone.0233620

**Published:** 2020-06-03

**Authors:** Heidi Albert, Ryan Purcell, Ying Ying Wang, Kekeletso Kao, Mathabo Mareka, Zachary Katz, Bridget Llang Maama, Tsietso Mots'oane

**Affiliations:** 1 FIND, Cape Town, South Africa; 2 LLamasoft Inc., St. Ann Arbor, MI, United States of America; 3 FIND, Geneva, Switzerland; 4 National Tuberculosis Reference Laboratory, Ministry of Health, Maseru, Lesotho; 5 National Tuberculosis Programme, Ministry of Health, Maseru, Lesotho; 6 Directorate of Laboratory Services, Ministry of Health, Maseru, Lesotho; Jamia Hamdard, INDIA

## Abstract

**Background:**

To reach WHO End tuberculosis (TB) targets, countries need a quality-assured laboratory network equipped with rapid diagnostics for tuberculosis diagnosis and drug susceptibility testing. Diagnostic network analysis aims to inform instrument placement, sample referral, staffing, geographical prioritization, integration of testing enabling targeted investments and programming to meet priority needs.

**Methods:**

Supply chain modelling and optimization software was used to map Lesotho’s TB diagnostic network using available data sources, including laboratory and programme reports and health and demographic surveys. Various scenarios were analysed, including current network configuration and inclusion of additional GeneXpert and/or point of care instruments. Different levels of estimated demand for testing services were modelled (current [30,000 tests/year], intermediate [41,000 tests/year] and total demand needed to find all TB cases [88,000 tests/year]).

**Results:**

Lesotho’s GeneXpert capacity is largely well-located but under-utilized (19/24 sites use under 50% capacity). The network has sufficient capacity to meet current and near-future demand and 70% of estimated total demand. Relocation of 13 existing instruments would deliver equivalent access to services, maintain turnaround time and reduce costs compared with planned procurement of 7 more instruments. Gaps exist in linking people with positive symptom screens to testing; closing this gap would require extra 11,000 tests per year and result in 1000 additional TB patients being treated. Closing the gap in linking diagnosed patients to treatment would result in a further 629 patients being treated. Scale up of capacity to meet total demand will be best achieved using a point-of-care platform in addition to the existing GeneXpert footprint.

**Conclusions:**

Analysis of TB diagnostic networks highlighted key gaps and opportunities to optimize services. Network mapping and optimization should be considered an integral part of strategic planning. By building efficient and patient-centred diagnostic networks, countries will be better equipped to meet End TB targets.

## Introduction

The World Health Organization (WHO) End tuberculosis (TB) Strategy calls for increased access to diagnostic tools for rapid and accurate detection of TB, universal access to drug susceptibility testing (DST), and strengthened quality of laboratory services [1], measures that will be critical to reaching national and global TB control targets. With an increasing choice of TB diagnostics endorsed by WHO for use in low and middle income settings [2], countries must select products that are appropriate for their own contexts and disease burdens, and design diagnostic algorithms to enable expanded access [3]. Lesotho, a small, land-locked, mountainous country in Southern Africa, is one of the 30 countries with the highest TB burden [4]. In 2017, over 15,000 cases of TB were reported (a rate of 665 per 100,000 population), of which 920 were multidrug resistant (MDR-TB); 73% of TB patients were co-infected with HIV [5]. Lesotho’s population of 2.1 million people is largely rural (72%), and the average per capita gross national income was only $1380 in 2018 [6]. The Ministry of Health (MOH) in Lesotho has rolled out the Xpert MTB/RIF test (Cepheid, Sunnyvale, CA, USA), a nucleic acid amplification test for the rapid diagnosis of TB and detection of rifampicin resistance. However, access to Xpert MTB/RIF testing and linkage of diagnosed patients to care remains a challenge [7].

Network design is the physical configuration and infrastructure of the diagnostic network, including the number, locations, and capacity of facilities and testing sites, and referral linkages. Network optimization, the selection of a best network configuration from a set of available alternatives based on selected criteria, can be used to inform instrument placement, sample transportation and referral mechanisms, staffing, geographical prioritization, quality assurance and integration of testing to meet the priority needs of a disease programme [8]. Network optimization and strategic supply chain management using specialized software and modelling approaches is common practice in the commercial sector [9]; while examples in the public health sector are somewhat limited, and mostly restricted to supply chain and procurement applications, modelling approaches have previously been used to inform placement of TB diagnostics in Tanzania [10], and of CD4 testing facilities in South Africa [11]. The latter included assessment of testing site workload, coverage areas and turnaround time to optimize service provision.

The patient pathway analysis (PPA) approach [12, 13] seeks to understand where patients seek care for TB and to align this with delivery of services. PPA has been conducted in five high burden TB countries [14–18] and has identified programmatic gaps in care seeking, screening, diagnosis, treatment and follow up. This study leveraged findings from PPA regarding where patients seek care, overlaid with data on availability and capacity for diagnostic testing, to map and model the existing network structure in Lesotho for TB diagnostic services using network design and optimization software. The study was intended to inform country-led decision-making processes around placement of new technologies and network optimization strategies aimed at achieving improved access to TB diagnostic services.

## Materials and methods

### Study setting

Lesotho comprises 10 administrative districts, and covers a total area of 30 355 km^2^ [19].

Lesotho’s health services are delivered at three levels, namely primary, secondary and tertiary levels. There were 286 public health facilities in Lesotho in 2016, including 265 primary health care centers, 20 general district hospitals, and a multi-specialty tertiary hospital (Queen Mamohato Memorial Hospital) located in Maseru. Patients requiring services beyond what is offered at the tertiary level are referred to hospitals in South Africa at the government’s expense. The Christian Health Association of Lesotho (CHAL) owns 8 of the district hospitals and 71 health centers while the rest are owned by government. Although CHAL is a private not for profit faith based organization, the CHAL facilities are operated as public facilities through funding provided by government [20]. In addition, a network of more than 6000 village health workers provides basic health services at community level [21].

Basic diagnostic services, including sputum smear microscopy and Xpert MTB/RIF testing, are largely available at district hospitals and some health centres, with specimens being referred from lower levels of the health system. Two regional referral laboratories and one National Tuberculosis Reference Laboratory in the capital, Maseru, provide specialised TB diagnostic services such as culture and drug susceptibility testing [21].

### Modelling process

**Fig 1:**
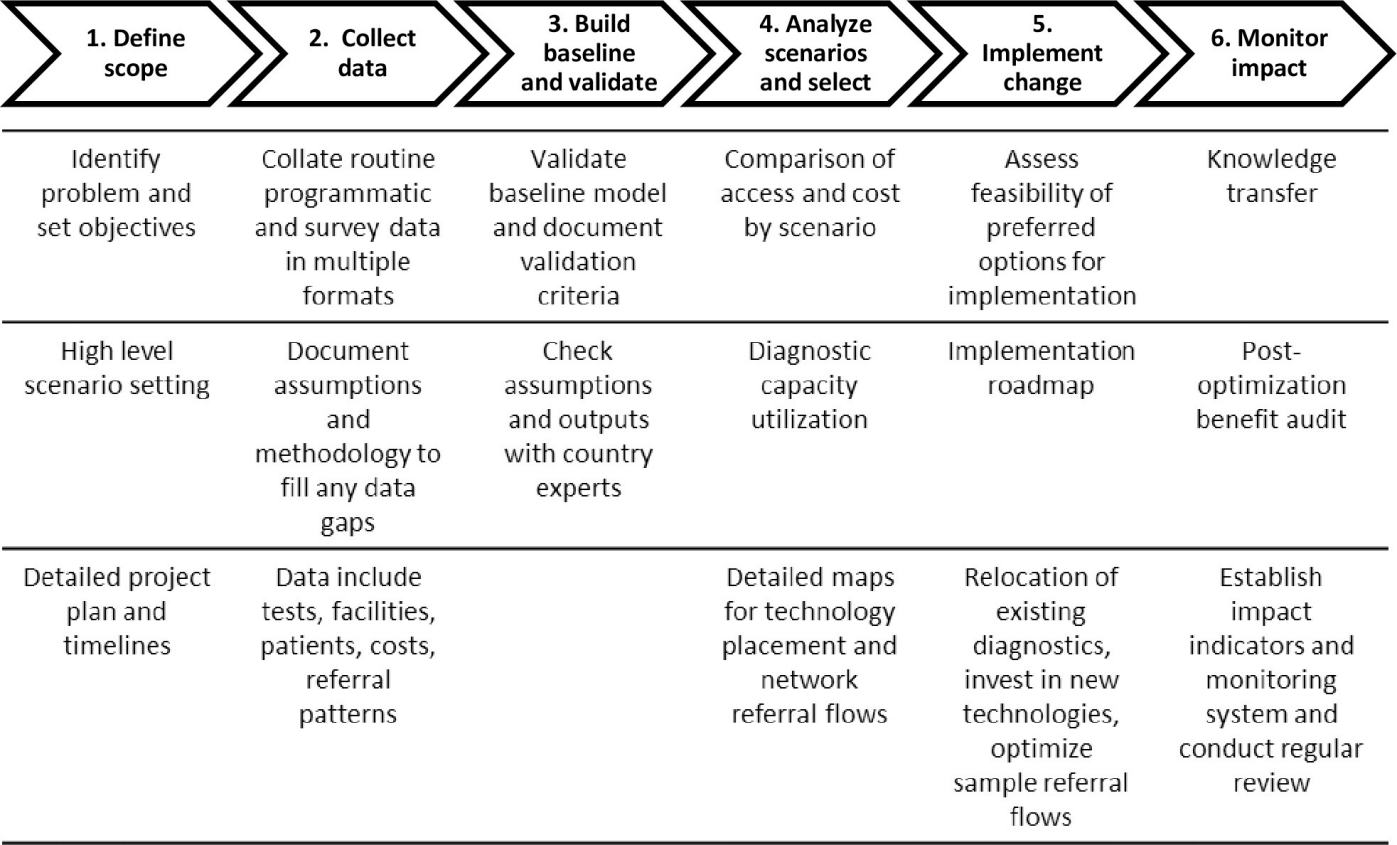
Steps in the diagnostic network optimization process.

Fig 1 outlines the steps involved in the Diagnostic Network Optimization process. The Ministry of Health and National Tuberculosis Programme (NTP) led the definition of the scope of the process, which was based on known challenges in delivery of TB services and need for data to inform planned interventions and investments in the TB diagnostic network. The analysis evaluated the status of the current diagnostic network, specifically the location and capacity of existing GeneXpert testing sites and how well this met current demand for services, as well anticipated increases in future demand as TB screening efforts are scaled up. Gaps in the diagnostic cascade for TB diagnosis were investigated as a means to identify opportunities to find missing cases and initiate patients on treatment, as well as to understand incremental increases in demand for testing as a result of closing gaps in the pre-testing part of the cascade (screening and referral of people with TB signs and symptoms for diagnostic testing).

Additionally, the process assessed whether existing plans for procurement of additional GeneXpert instruments were necessary and if so, which would be the optimal locations for their placement. Lastly, the impact of placement of new point-of-care (POC) devices that are expected to be available in the near future, such as the GeneXpert Omni device, on access to services and cost of the diagnostic network, was evaluated.

### Baseline model structure and data inputs

#### Methodology

The TB diagnostic network structure was modelled using a commercially available software tool, Supply Chain Guru® (LLamasoft, Inc, Ann Arbor, MI). This software tool leverages a Mixed Integer Linear Program (MILP) to analyze and optimize the supply chain network [22]. Locations for testing demand (patients) and testing supply (diagnostic devices) are incorporated, along with the sourcing and transportation pathways connecting patient test demand to diagnostic testing sites, including the costs and lead times associated with each pathway. In addition, instrument costs and capacity constraints are included to ensure the results of the model accurately represent the realities of operating the diagnostic testing services within the network. Scenarios (see Table 2) are then created to test out various future state configurations for the network. The resulting ‘optimized’ solution is the lowest cost solution that meets all constraints imposed upon the network model. The analysis largely focused on estimating the extent and distribution of diagnostic capacity that would be needed in the country under various demand scenarios. As such the costing approach focused on costing different sample transport routes that would be considered in the different scenarios (see below for transportation costing methodology). No detailed route planning or optimization was conducted in this study. The model output was therefore the network design that gave the lowest transportation cost at each demand level.

#### Data sources

Data sources used to populate the model are shown in Table 1. These files were reviewed for completeness, plausibility and internal consistency of inputs, and were confirmed with stakeholders, before being merged and formatted into the software’s model database schema.

**Table 1:** Methods and data sources used to estimate each step of the TB diagnostic cascade for patients in Lesotho in 2015.

Stage	Number of persons	% of previous stage	Data source
Population	2,160,309	-	Lesotho Bureau of Statistics, 2015. [23] Health facility catchment population data, gender disaggregated, Ministry of Health Lesotho (unpublished data)
Total prevalent TB cases (all forms)	16, 700		WHO Global TB Report, 2016 [24]
Total notified TB cases (all forms)	7, 758		NTP 2016 Report (unpublished data)
Persons screened for TB (verbal symptom screen by VHWs in community or at health facilities)	1,125,486	52%	NTP 2016 Report
Persons screened who were found to have TB signs and symptoms^1^	41,189	4%	NTP 2016 Report
Persons examined with Xpert MTB/RIF test	29,913	73%	National TB Reference Laboratory, 2015
Persons diagnosed with bacteriologically-confirmed pulmonary tuberculosis (drug susceptible and drug resistant TB)	3,435	11%	NTP 2016 Report
Persons diagnosed with bacteriologically-confirmed pulmonary tuberculosis who started treatment	2,806	82%	NTP 2016 Report

TB–tuberculosis, VHW–village health worker.

^1^ TB signs and symptoms include cough of two weeks or more, weight loss, fever and night sweats

NTP–National Tuberculosis Programme.

**Fig 2:**
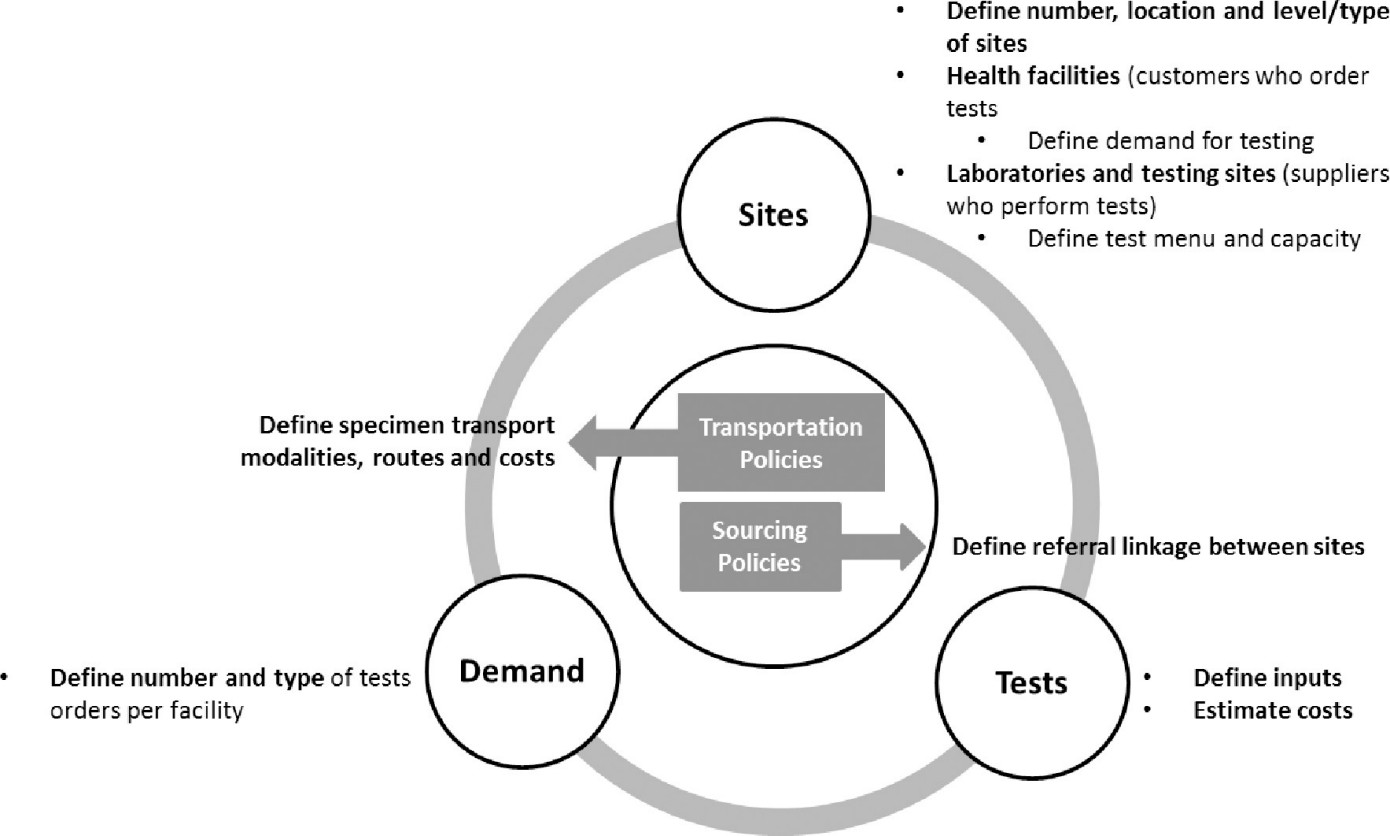
Diagrammatic representation of the data inputs required for diagnostic network optimization.

Data inputs required for optimization of the model are shown in Fig 2. Due to sub-national variations, facility or district level data were used where available. Sites included health facilities that order diagnostic tests (referring facilities) and facilities with laboratories and/or testing sites that conduct tests (referral facilities). Some sites included both referring and referral capacity, e.g. health facilities that conduct smear microscopy but refer specimens to a higher-level facility for Xpert MTB/RIF or culture testing. The analysis primarily focused on Xpert MTB/RIF testing sites and therefore data on other tests (smear, culture and other DST) were excluded from the detailed analysis.

#### Demand estimation

National demand for testing was estimated at three levels: baseline (current demand), intermediate demand, and total demand. Current demand for testing was the number of Xpert MTB/RIF tests conducted in 2015 per referring facility as communicated by NTP/national TB reference laboratory (NTRL); 30,000 tests/year (Mareka, M, personal communication. Intermediate demand was estimated from diagnostic cascade data, as the number of Xpert MTB/RIF tests that would be performed if all people who were screened for TB and found to have signs and symptoms were tested (41,000 tests/year). The total demand for testing was defined as the number of Xpert MTB/RIF tests that would be required to find all TB cases in the country (including intermediate demand [those screened with signs and symptoms] as well as testing required for additional screening; 88,000 tests/year). To provide context to the total demand estimate, the overall projected demand of 88,000 tests equates to all PLHIV (constituting approximately 15% of the country’s population in 2016 [25]) being screened two to three times a year, and the rest of the general population being screened once a year. The number of TB patients per health facility was estimated based on the age and gender disaggregated catchment population per facility (Ministry of Health Lesotho, unpublished data) and the 2015 TB incidence data reported by WHO [26]. The unmet demand for TB diagnostics was further split into (a) unmet demand among those screened for TB (patients who have already entered the health system, and (b) unmet demand among those unscreened (patients who have not entered the health system).

#### Sample transport and linkages

Different sample referral and transportation systems were employed at different levels within the network. Riders for Health is a non-governmental organisation that provides sample transportation services from level 1 (health centres or clinics) to district hospitals (level 2) for diagnostic testing. For referral of samples from district hospitals to NTRL, a courier company is contracted. *Ad hoc* transportation is done via hospital or other transport, e.g. during site supervision visits by regional or national level staff. For estimating distances between all level 0 and any level 1 or 2 facilities, actual distances were used where available (for existing transportation routes). Where actual distances were not known, a distance adjustment factor was computed from actual distance divided by the straight-line distance on the map for the lanes where actual distances exist and applied to unknown transport lanes based on the source and destination districts. For estimating unknown actual distances, the straight-line distance was multiplied by the average transport adjustment factor.

Data on linkages between health facilities and testing sites at baseline were provided by Riders for Health and the MOH. For network optimization scenarios, we evaluated new linkages among all health facilities and all testing sites in the model to potentially improve network efficiency and find the optimal network configuration and equipment placement. Any proposed linkages from the model solutions were then subjected to validation and assessment of feasibility for implementation in consultation with NTP/MOH staff.

Courier costs for shipment of samples from district hospitals to NTRL were flat rates per trip and not distance-based, and were therefore excluded from the analysis since they do not impact on the model’s decisions on where to place GeneXpert instruments. For transportation of samples from health facilities and clinics to district hospitals, an average cost per sample transported per kilometre was calculated for each district using historical cost data (Riders for Health, unpublished data), and then applied to transportation routes in the new scenarios. The actual transport cost per district was provided by Riders for Health, which included fuel, administrative costs, maintenance and training costs, as well as *ad hoc* staff that were engaged in addition to regular motorbike riders. Data on the number of riders per district and their salaries in 2015 were also provided by Riders for Health. The fuel cost was estimated at 0.09 US Dollars per kilometre. The following assumptions were applied: each rider conducted one trip per day and travelled at an average of 30km/hour, there were 240 working days per year and three samples were transported for each presumptive TB patient undergoing investigation (two samples for smear microscopy and one for Xpert MTB/RIF testing).

### Evaluating the baseline and alternative network models

We analysed a range of alternative network scenarios based on the existing network structure to assess future planned procurements of instruments, alternative possible structures utilizing the GeneXpert Omni device (under development), as a proxy for point-of-care tests for rapid TB detection, that would be implemented in conjunction with existing technologies, and scenarios that enabled unconstrained placement of instruments at existing or new locations (Table 2). Outputs of scenarios were validated and assessed for feasibility.

**Table 2:** Descriptions of diagnostic network scenarios and assumptions used in constructing network models and running optimization scenarios.

	Description	Assumptions
Baseline	Existing locations and active GeneXpert capacity as of January 2017	
Network 2017	As baseline, with 13 additional GeneXpert instruments operational at their assigned locations	13 GeneXpert instruments were procured in 2016. They were non-operational at the time of the study due to lack of human resources.
Optimal 13–20 sites	As baseline, with placement of 13 additional GeneXpert instruments being optimized. Up to 7 new GeneXpert instruments can be added and their locations optimized.	Assumes location of 13 GeneXpert instruments could be reconsidered by MOH. Funding for a further 7 GeneXpert instruments had been allocated but orders not yet placed.
Network 2018	As Network 2017. Additional 7 GeneXpert instrument added to network in optimal locations.	Assumes location of 13 GeneXpert instruments can not be reassigned, and that procurement of a further 7 GeneXpert instruments proceeds in 2017.
Optimal GX + Omni	As per Optimal 13–20. Any number of additional GeneXpert instruments or Omni instruments can be added to the network at their optimal locations.	Unconstrained addition of GeneXpert and/or Omni capacity to existing optimized network design.

## Results

### Current TB diagnostic network status

**Fig 3:**
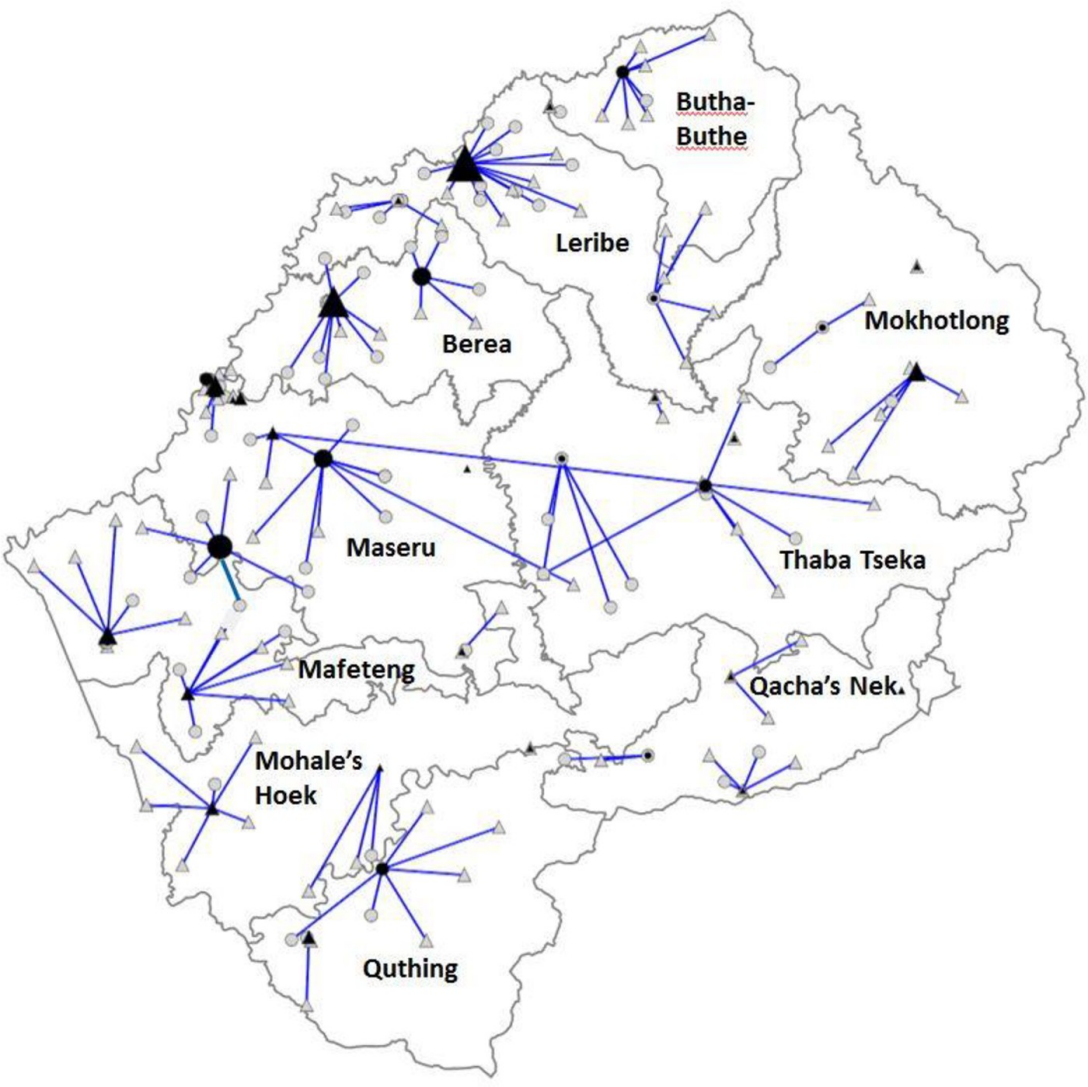
Map of Lesotho’s TB diagnostic network structure, 2016. Public sector facility; triangle; private sector facility; circle. Size of facility icons (black) are scaled according to Xpert MTB/RIF testing volume in 2015.

**Fig 4:**
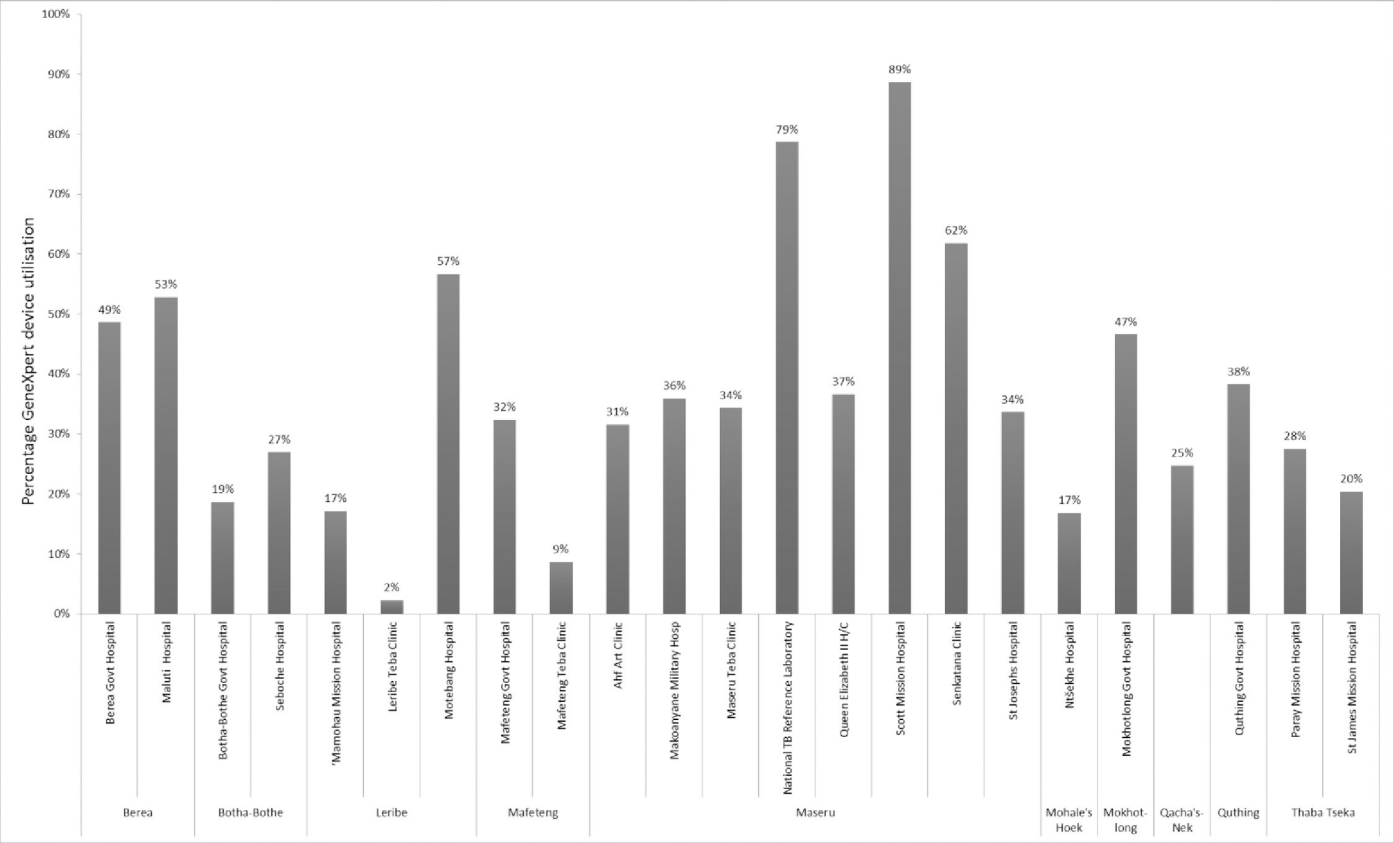
Utilization of GeneXpert device capacity per testing site and district, 2016. Maximum capacity per GeneXpert four-module device was considered as 12 tests per day for 240 working days per year (total of 2880 tests per year).

Fig 3 shows the network structure in 2016. Utilization of GeneXpert instruments was found to be low (19/24 testing sites use less than 50% of instrument capacity), and varied considerably across sites, with utilization rates varying from 89% to 2% (Fig 4). The capacity and location of GeneXpert instruments in the network was compared with current, intermediate and total demand for testing. The current network had sufficient GeneXpert capacity to meet both existing and intermediate demand, and could manage approximately 70% of the total demand.

The testing capacity within the network was generally well-positioned when comparing the current location of testing capacity with the optimized network structure generated by the software analysis. However, the analysis predicted that relocation of certain instruments could improve overall network efficiency by moving additional capacity closer to where patients seek care. Specifically, the modelling suggested that the locations selected for placement of 13 GeneXpert instruments that were procured in 2016 but were not yet operational at the time of the study are not all optimal. Relocation of 6 out of the 13 instruments would improve network performance. The extent of network optimization possible through relocation of these instruments produced an equivalent effect to procurement of 7 further GeneXpert instruments.

### TB diagnostic cascade

**Fig 5:**
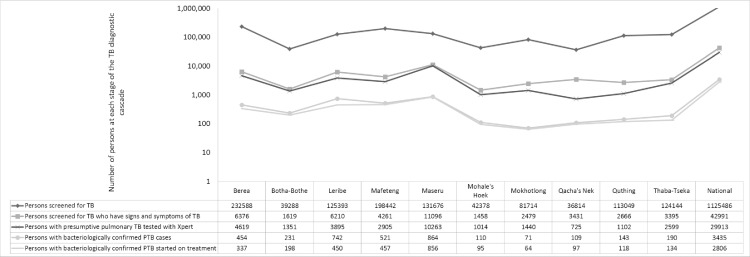
TB diagnostic and treatment cascade in Lesotho, 2016 (national and by district). TB–tuberculosis, PTB–pulmonary tuberculosis.

Fig 5 shows the steps in the TB diagnostic cascade and the number of people at each step, both at a national level and per district. The extent of screening events varied substantially across districts, with the number of screening events equating to 22% of the population in Mohale’s Hoek to 97% of the population in Mafeteng. However these figures do not account for repeated screening of individuals, e.g. PLHIV, and are therefore not reflective of the proportion of the population who are screened. Similarly the yield of TB symptomatic individuals among those screened varied from 2.1% in Mafeteng to 8.4% in Maseru. Linking people who were screened and found to have TB signs and symptoms to Xpert MTB/RIF testing varied from 41% in Quthing to 92% in Maseru. Closing this gap would need an additional 11,000 tests overall, and result in approximately 1000 additional TB patients being treated.

Xpert MTB/RIF test positivity varied from 5% in Mokhotlong to 19% in Leribe. The percentage of diagnosed patients who started TB treatment varied from 61% in Leribe to 99% in Maseru.

Closing this gap would lead to an additional 629 TB patients being treated.

### Future demand

The current network can meet about 70% of the estimated total demand needed to find all TB cases (approximately 60,000 of the 88,000 tests needed per year). Factors that were important when evaluating overall network performance as demand increases included increasing utilization, percentage of onsite testing turnaround times and transportation costs. As compared with baseline, the network 2017 scenario (which includes an additional 13 GeneXpert devices) has a lower utilisation rate, a higher proportion of on-site testing (45% compared with 33%), slightly reduced turnaround time (3.7 days compared with 4.0 days) and transportation cost estimate reduced by approximately one third. As testing demand increases, the average utilisation of instruments increases (to 35% and 76% at intermediate and total demand level), fewer samples are tested on-site as demand increases (45% at current demand, 39% at intermediate demand and 24% at total demand level), turnaround time at intermediate demand is similar to baseline but increases at the total demand level by approximately 1 day compared to baseline. Transportation costs at intermediate demand level for the network 2017 design was comparable to baseline, with an increase by a factor of 30% with the total testing demand estimation.

Screening was the main driver of demand; 75% of the unmet demand (additional volume of tests required to find the missing TB cases) came from people who were never screened.

The model suggested that total demand for testing could be met with no additional GeneXpert instruments (assuming locations are optimized) and 33 point of care instruments (such as the Omni device under development). The cost of 33 point of care instruments was comparable to that of 7 GeneXperts, using the cost assumptions available at the time of the study.

## Discussion

The findings of this study resulted in a number of recommendations for the Lesotho TB diagnostic network. The pre-study decision to procure an additional seven GeneXpert instruments was shown to be unnecessary, as comparable benefits could be obtained by relocating existing devices. This would allow funding allocated for new instruments to be moved to other priority areas, including sample transportation, supervision and quality assurance, and equipment maintenance, which are often under-funded and yet are essential components of a quality-assured diagnostic network. While no major changes in network structure were required, minor changes in location of instrument capacity to bring GeneXpert instruments closer to demand for testing could result in improved access, reduced transport costs and improved turnaround times. Flexibility to relocate instruments should be considered as a mechanism to improve network efficiency and avoid unnecessary expansion of testing sites, reduce capital expenditure and recurrent resources (financial and human) that are needed to maintain quality-assured service delivery. Instrument placement decisions are frequently influenced by geographical placement or other considerations, but as demonstrated by our model, the focus should be on placement of technology close to where patients seek care, and near high-risk populations.

This analysis highlighted two key areas in which demand for testing and impact of testing could be strengthened. The first relates to linking of people who were screened and found to have TB signs and symptoms with Xpert MTB/RIF testing, to ensure confirmation of diagnosis. Significant variation was observed across districts, warranting an in-depth assessment to understand the gaps between screening and testing, and to facilitate the development of successful strategies to close these gaps. The second area related to linking confirmed TB patients to treatment. Identification of best practices may help with scale up successful strategies to address these gaps. Notably, the GeneXpert positivity rate was variable across testing sites, highlighting a need for understanding of the drivers of GeneXpert test positivity in relation to the population screened and referred for testing and how this may vary across the country. An assessment of laboratory quality indicators and systems to determine whether variability is related to testing practices is recommended.

Analyses of future demand showed that an immediate scale up of laboratory services is not warranted, as significant increases in demand could be met with current structure and testing capacity. However, scale up and optimization of the lab network would be needed in order to find all of the TB cases in Lesotho. This would be highly reliant on parallel and aligned scale up of screening and treatment services. Since the volume and effectiveness of screening is the main driver for TB testing demand, alignment of plans for screening scale up with laboratory capacity is critical. Proactive screening of individuals in “hotspot” areas and referral of those identified as presumptive TB patients for testing are important interventions, requiring strong community-based outreach and advocacy measures to mobilise the population. Understanding the population’s care seeking behaviour and ensuring availability of services aligns with where patients seek health care services is critical. Furthermore, as Lesotho embarks on plans for decentralized viral load/early infant diagnosis testing (VL/EID) for HIV, which will also be performed using the GeneXpert platform, it will be important to update the network analysis to account this and ensure sufficient capacity for both scale up of TB testing and for the HIV programme. Further analyses to take into account more granular input data relating to costs and updated costing assumptions, human resources and operational considerations associated with implementation of the Omni instrument would also be of value.

In the two years since this study was conducted the Lesotho GeneXpert network has been expanded with the placement of 24 new instruments in existing sites and the opening of two new GeneXpert testing sites. TB testing has also been integrated with HIV VL/EID testing in 12/24 new sites and two new sites provide only HIV VL/EID testing. Although none of the instruments were relocated to improve network efficiency as recommended by the model, interventions being implemented by the TB programme have resulted in an increase in demand for TB testing that already exceeds estimated intermediate demand levels. Instrument utilization rates have also increased and in the period of January to June 2019, a total of 35,435 TB tests were run on the existing network with the rest of the capacity being utilized for HIV VL/EID testing in the integrated laboratories. In eight laboratories the utilization rate was still below 50% of capacity indicating areas for potential improvements in efficiencies. In 2019/2020 Lesotho will be undergoing a further network optimization exercise that looks at optimizing the diagnostic network to deliver integrated TB/HIV diagnostic services. This expanded network optimization work builds on the experience gained from the network optimization exercise and recommendations described herein. Diagnostic network design and optimization analysis [27] has also subsequently been undertaken in Kenya [28] and the Philippines [29], with outputs informing development of Kenya’s National Strategic Plan for Tuberculosis Leprosy and Lung Health [30], informing county level sample referral network design and operational planning, and informing placement of additional GeneXpert testing capacity in the Philippines.

Limitations of this study included challenges experienced with data quality (lack of internal completeness and consistency across different data sources), particularly with relation to screening results. Improving quality of routine data is a priority as countries move towards data-driven decision-making. Subsequently, after this analysis was performed, NTP introduced a standardized screening tool and engaged data clerks at health facilities to capture and report data. Challenges in implementing the findings of the diagnostic network optimization exercise included the critical nature of timing to ensure that the model can be utilized to inform decision-making around funding and investment decisions. For example, decisions around instrument placement, particularly where this requires any infrastructure investment, need to be made well in advance. Multiple partners or donors may be involved in support of diagnostic networks, and therefore their planning and budgeting processes need to be taken into account and they must be engaged early on in the network modelling and optimization process to ensure the outputs fit their needs.

## Conclusions

In summary, this analysis demonstrated that the current TB diagnostic network in Lesotho has sufficient capacity to meet current demands, but highlighted key gaps and opportunities to improve access to services, which will need to be acted upon in order to meet the total demand for TB testing in the country. Use of this data-driven approach to network design is recommended to other countries, to understand how best to improve access to rapid TB diagnosis and DST, to increase the efficiency of service delivery to meet the needs of patients and to move towards meeting national and global TB targets.

## Supporting information

S1 File Baseline customer demand for steps in the TB diagnostic cascade in Lesotho, 2016. (XLSX)

S2 File Facility list for existing and potential sites, Lesotho TB diagnostic network. (XLSX)

S3 File Outputs of optimized scenarios, Lesotho 2017. (XLSX)

S4 File Unmet demand estimation per district. (DOCX)

S5 File Sample transportation costs, 2016. (DOCX)
