# Supplementary material for: Designing an optimized diagnostic network to improve access to TB diagnosis and treatment in Lesotho
Source: PLoS One. 2020 Jun 3;15(6):e0233620. doi: 10.1371/journal.pone.0233620 (PMC7269260; doi:10.1371/journal.pone.0233620)
Supplement: S4 File — (DOCX) [file pone.0233620.s004.docx]

1. **Unmet demand estimations per district, Lesotho**

PTB, pulmonary tuberculosis; EPTB, extra-pulmonary tuberculosis
