# Supplementary material for: Designing an optimized diagnostic network to improve access to TB diagnosis and treatment in Lesotho
Source: PLoS One. 2020 Jun 3;15(6):e0233620. doi: 10.1371/journal.pone.0233620 (PMC7269260; doi:10.1371/journal.pone.0233620)
Supplement: S5 File — (DOCX) [file pone.0233620.s005.docx]

1. **Estimation of Sample Transportation Costs, by district, 2016**

| **District** | **Total cost**  **(US Dollars)** | **Estimated distance of sample transport routes (km)** | **Total samples transported** | **Cost per shipment per km** | **Number of motorbike couriers** | **Shipment size** | **Cost per sample per km** |
| --- | --- | --- | --- | --- | --- | --- | --- |
| Maseru | 131, 130 | 255, 744 | 188, 346 | 0.513 | 12 | 65 | 0.008 |
| Leribe | 65, 565 | 127, 872 | 86, 339 | 0.513 | 6 | 60 | 0.009 |
| Berea | 32, 783 | 63, 936 | 53, 215 | 0.513 | 4 | 55 | 0.009 |
| Botha-Bothe | 31, 247 | 47, 952 | 38, 367 | 0.652 | 3 | 53 | 0.012 |
| Mokhotlong | 32, 783 | 63, 936 | 29, 555 | 0.513 | 3 | 41 | 0.012 |
| Mohales Hoek | 31, 247 | 63, 936 | 22, 253 | 0.489 | 3 | 31 | 0.016 |
| Quthing | 43, 710 | 63, 936 | 23, 818 | 0.684 | 3 | 33 | 0.021 |
| Thaba Tseka | 41, 670 | 63, 936 | 29, 833 | 0.652 | 4 | 31 | 0.021 |
| Mafeteng | 41, 663 | 47, 952 | 17, 015 | 0.869 | 3 | 24 | 0.037 |
| Qacha’s Nek | 32, 783 | 85, 248 | 7, 283 | 0.385 | 4 | 8 | 0.051 |
